# Bibliometric Analysis of Information Theoretic Studies

**DOI:** 10.3390/e24101359

**Published:** 2022-09-25

**Authors:** Weng Hoe Lam, Weng Siew Lam, Saiful Hafizah Jaaman, Pei Fun Lee

**Affiliations:** 1 Department of Physical and Mathematical Science, Faculty of Science, Universiti Tunku Abdul Rahman, Kampar Campus, Jalan Universiti, Bandar Barat, Kampar 31900, Perak, Malaysia; 2 Department of Mathematical Sciences, Faculty of Science and Technology, Universiti Kebangsaan Malaysia (UKM), Bangi 43600, Selangor, Malaysia

**Keywords:** information theoretic, bibliometric analysis, subject area

## Abstract

Statistical information theory is a method for quantifying the amount of stochastic uncertainty in a system. This theory originated in communication theory. The application of information theoretic approaches has been extended to different fields. This paper aims to perform a bibliometric analysis of information theoretic publications listed on the Scopus database. The data of 3701 documents were extracted from the Scopus database. The software used for analysis includes Harzing’s Publish or Perish and VOSviewer. Results including publication growth, subject areas, geographical contributions, country co-authorship, most cited publications, keyword co-occurrence analysis, and citation metrics are presented in this paper. Publication growth has been steady since 2003. The United States has the highest number of publications and received more than half of the total citations from all 3701 publications. Most of the publications are in computer science, engineering, and mathematics. The United States, the United Kingdom, and China have the highest collaboration across countries. The focus on information theoretic is slowly shifting from mathematical models to technology-driven applications such as machine learning and robotics. This study highlights the trends and developments of information theoretic publications, which helps researchers to understand the state of the art of information theoretic approaches for future contributions in this research domain.

## 1. Introduction

Entropy function is the basic building block of information theory. The calculation of entropy for a probabilistic system is widely recognized as the standard way to quantify the amount of uncertainty in that probabilistic system [1]. Statistical information theory provides a framework to quantify in a single value the proportion of total information in one set of measures explained by another set of measures and also quantifies the amount of redundant information.

The application of information theoretic approaches has been extended to various areas. Information theoretic approaches have been applied in the analysis of questionnaire data [2], product vulnerability [3], and brand switching [4]. Besides that, the information theoretic approach has been illustrated for identifying shared information and asymmetric relationships among variables [5]. Still and Precup [6] have studied reinforcement learning by drawing on ideas from information theory, and information theoretic approaches have been studied by past researchers in stock markets and complex systems [7,8,9,10,11].

The bibliometric analysis involves systematic quantitative and qualitative analyses of a particular research title or domain to assess the publication trends and developments from time to time [12]. The main advantage of bibliometric analysis is the ability to assess publication growth, geographical contribution, research focus, and scientific achievements such as citations and h-index [13]. Popular analyses in bibliometrics include publication growth, subject area, geographical contribution, source title, most cited publications, and keyword analyses [14].

Bibliometric analysis helps researchers to identify emerging areas and future directions of the research domain with the help of visualization tools. Bibliometric analysis is able to handle massive volumes of unstructured data from scientific databases and provide factual and objective information in the form of citation metrics [15]. According to our best understanding, there has been no past bibliometric analysis of information theoretic, therefore, this paper aims to perform a bibliometric analysis of information theoretic based on documents indexed in the Scopus database from 1958 to 2022. This bibliometric paper aims to provide a comprehensive overview of information theoretic studies and help researchers derive groundbreaking ideas by looking at the knowledge gaps.

This paper continues as follows. Section 2 introduces the data and methodology. Section 3 presents the bibliometric analysis results, including the publication trends, subject areas, geographical contribution, country co-authorship, famous source titles, most cited publications, keyword analyses, and citation metrics, and Section 4 concludes the paper with a summary and limitations of the study.

## 2. Data and Methodology

**Figure 1:**
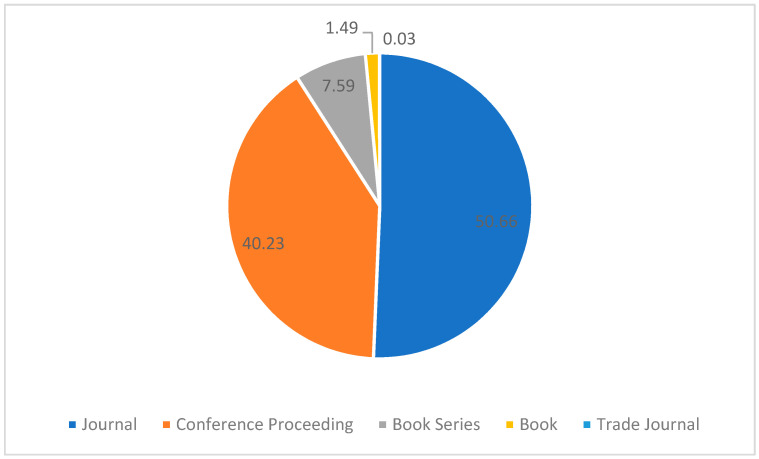
Source types.

This study assesses the literature of information theoretic with data obtained from the Scopus database. Scopus is a universally accepted citation database that houses many high-quality peer-reviewed papers [16,17]. Since Scopus has wider coverage than the Web of Science, it is highly suited to be used in bibliometric analysis [18,19]. Data mining was performed from the Scopus database on 17 August 2022. This study focuses on “information theoretic” in the title. The first publication was made in 1958 and there have been 3701 papers as of 17 August 2022. The following search query was used: (TITLE (“information theoretic”)) and a total of 3701 documents were obtained from this query. The 3701 publications were classified into eleven document types, as shown in Table 1. The two most popular document types were conference papers (1759) and articles (1756), accounting for 47.53% and 47.45% of the total publication, respectively. Other document types included book chapter (72), review (51), erratum (18), conference review (14), editorial (10), letter (10), note (6), book (4), and data paper (1). Table 1 lists the frequencies and percentages of the document types of the 3701 publications under information theoretic. The source type is the final published version of a document; out of the 3701 documents, 50.66% of the documents were published in journals (1875), 40.23% of the papers were published in conference proceedings (1489), and the remaining documents were published in book series (281 documents or 7.59%), books (55 documents or 1.49%) and trade journals (1 document or 0.03%). Figure 1 describes the source types of information theoretic publications. All the data used in this bibliometric analysis were downloaded on 17 August 2022.

Upon extracting the data from the Scopus database, Harzing’s Publish or Perish 8 was used to process and analyze the information. The strength of bibliometric analysis revolves around its ability to provide powerful quantitative and qualitative indicators of the title under study. The total number of papers (TP) is an important quantitative index to measure publication growth over the years. Other indices such as total citation (TC), number of cited papers (NCP), citation per paper (C/P), and citation per cited paper (C/CP) contribute to the qualitative assessment in bibliometric analysis. For future publications forecasting, h-index (h) and g-index (g) are often studied together. All these indicators, based on publication growth, country, and source title, can be obtained from Harzing’s Publish or Perish software [20,21,22].

Network visualization and overlay visualization can be mapped with VOSviewer. VOSviewer is an open-source programme created by Van Eck and Waltman [23]. VOSviewer has also gained popularity due to its ability to perform co-occurrence and co-citation analyses with a user-friendly interface for map generation [24]. In this study, VOSviewer version 1.6.17 is used to visualize keyword co-occurrences and country co-authorship.

## 3. Results

This section covers the bibliometric analysis of information theoretic based on data extracted from the Scopus database. Publication growth, subject areas, geographical contributions, highly cited publications, and keyword analyses will be performed in this section. In the end, citation metrics will also be presented for a brief summary of all the 3701 publications under information theoretic, as of 17 August 2022.

### 3.1. Publication Growth

The annual publication of information theoretic papers from 1958 to 2022 is presented in Table 2. The publication growth of information theoretic papers was slow from the first paper published in 1958 until 2002. The average annual growth rate in this period was approximately 20%. The number of published papers exceeded 100 in 2003 and has been relatively stable since then, as there has been an increase in popularity among researchers towards information theoretic. Quantifying information based on probability distribution is necessary for more data-driven analyses. Information theoretic has attracted massive attention from computer science, engineering, and mathematics researchers. Since data extraction from the Scopus database was performed on 17 August 2022, the TP in 2022 was still unavailable. However, 96 papers have been published and indexed in Scopus database from 1 January to 17 August 2022. The citation metrics of the annual information theoretic publications are also listed in Table 2. The highest C/P was found in 1985 as there were 2934 total citations from only 16 publications, yielding a C/P index of 183.38 and a C/CP index of 244.50 because there were 12 cited papers. The high C/P and C/CP indices in 1985 were contributed by the highest cited publication by Wax and Kailath [25]. This paper has received 2731 citations since its publication.

### 3.2. Subject Areas

Information theoretic has attracted massive attention from researchers in the computer science, engineering and mathematics areas. Information theoretic adopts mathematical laws to monitor and maximize the value of data movement, storage, and retrieval [26]. In total, 2259 papers (32.89%) were listed under computer science, while 1254 papers (18.26%) and 1068 papers (15.55%) were categorized under engineering and mathematics, respectively. Other areas that apply information theoretic include physics and astronomy (8.36%), social sciences (3.61%), materials science (2.77%), biochemistry, genetics and molecular biology (2.66%), chemistry (1.86%), medicine (1.86%) and decision sciences (1.76%). The complete list of subject areas associated with information theoretic is presented in Table 3.

### 3.3. Geographical Contribution of Information Theoretic Publications

Information theoretic has attracted the contributions of researchers from more than 80 countries worldwide. The United States (1624) contributes the highest to the TP, followed by China (285) and the United Kingdom (279). The total citation from the 3701 publications is 82,000. Publications from the United States have received 45,797 citations, which amounts to 55.85% of the total citation. The country with the second highest number of citations is the United Kingdom, with 7352 citations.

Meanwhile, the United States also has the highest h-index of 89. This means that 89 papers have been cited at least 89 times, signaling high scientific achievement [21]. The United Kingdom and Germany have an h-index of 40 and 35, respectively. The highest C/P and C/CP are 41.58 and 49.46, respectively, and are contributed by Israel. Even though China has the second highest number of publications, China still needs to improve the quality of its scientific contributions. Table 4 presents the top 10 geographical contributions of information theoretic publications.

**Figure 2:**
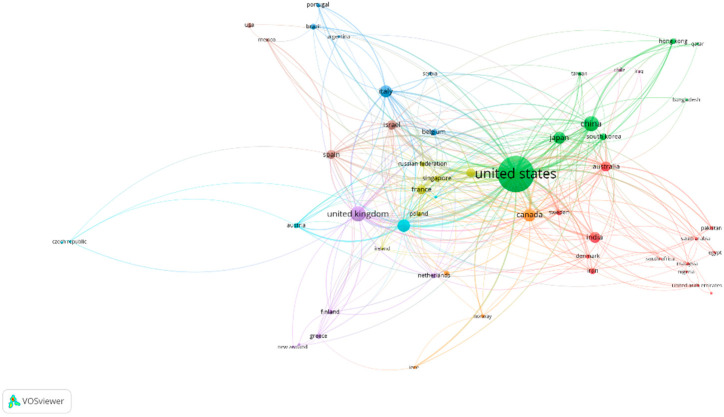
Country co-authorship network.

In many publications, there is more than one researcher due to the complexity and scope of the study, which requires collaboration; co-authorship analysis assesses this research collaboration. Country co-authorship analysis portrays the level of collaboration among countries and highlights the most prominent countries in the research domain. The country co-authorship network map for information theoretic publication is shown in Figure 2. The larger node represents more prominent countries in the research interest of information theoretic. The length between the nodes and the boldness of lines reflect the collaboration among researchers across countries. From Figure 2, the most prominent countries active in information theoretic publications co-authorship are the United States, United Kingdom, China, Germany, Canada, France, Italy, Israel, Japan, and Australia. The United States has the highest total link strength of 490 from more than 1600 publications, followed by the United Kingdom (193), China (169), Germany (128), and Canada (127).

### 3.4. Source Titles of Information Theoretic Publications

Table 5 presents the top 10 source titles that published information theoretic documents. Lecture Notes in Computer Science, including Subseries Lecture Notes in Artificial Intelligence, and Lecture Notes in Bioinformatics published 221 documents, followed by the IEEE International Symposium on Information Theory Proceedings (139) and IEEE Transactions on Information Theory (103).

### 3.5. Most Cited Information Theoretic Publications

Table 6 tabulates the top 10 most cited publications in information theoretic since 1958. The most cited document, “Detection of signals by information theoretic criteria” by Wax and Kailath [25], received 2731 citations and 73.81 citations per year. This paper proposed a new method to detect the amount of signal in a multi-channel time series, removing any subjective setting. The second most cited paper was “Breaking spectrum gridlock with cognitive radios: an information theoretic perspective by Goldsmith et al. [27]. This paper discussed the use of cognitive radios for higher spectral efficiency with an information theoretic survey. The third most cited paper by Biglieri et al. [28] found that information theoretic approaches such as equalization, coding, and modulation enhance the performances of fading dispersive channels. Davis et al. [29] introduced an information theoretic method for learning a Mahalanobis distance function. This study showed that the proposed method could handle multiple constraints and incorporate a prior on the distance function. The following most cited paper by Bloch et al. [30] focused on the transmission of confidential data over wireless channels. This paper developed a practical secure communication protocol to ensure wireless information-theoretic security.

Pedersen [31] presented an efficient non-interactive scheme for verifiable secret sharing without information about the secret. Vinh et al. [32] presented an organized study of information theoretic measures for comparing clusterings. The findings showed that the normalized information distance and normalized variation of information satisfy the normalization as well as metric properties. The next most cited paper by Dhillon et al. [33] proposed an innovative co-clustering algorithm to increase the preserved mutual information by intertwining the column and row clusterings at all stages. This paper showed that the proposed algorithm performed well, especially in the presence of high-dimensionality and sparsity. Ozarow et al. [34] presented an information theoretic analysis associated with digital cellular radio that focused on time division multiple access protocols. Based on the information theoretic point of view, double ray propagation was advantageous over a single ray propagation when both normalized to the same power. The tenth most cited paper by Brown et al. [35] proposed a unifying framework for information theoretic feature selection. This paper showed that common heuristics for information-based feature selection are approximate iterative maximizers of the conditional likelihood.

### 3.6. Keyword Analyses

**Figure 3:**
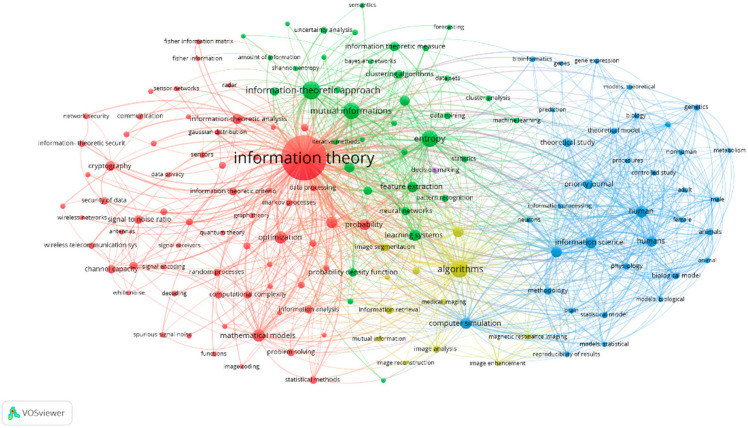
Keyword co-occurrence map.

This subsection includes various keyword analyses such as the keyword co-occurrence network and keyword overlay visualization maps. Keyword co-occurrence provides research highlights of the title under study. In all the 3701 documents, there are 16,828 indexed keywords as furnished by VOSviewer. One of the advantages of VOSviewer is the ability to adjust the minimum occurrences that the keywords should achieve in the visualization maps for better interpretation, clarity, and understanding. The frequency with which a keyword appears indicates its significance in the study domain. The more the keyword appears among the 3701 publications, the greater the attention placed on the research area based on the keyword, and this would also create a map with high clarity for better analysis [36,37,38]. Therefore, the keyword co-occurrence map in Figure 3 considers only keywords that have appeared more than 30 times, with 148 keywords matching the threshold and relevant to the information theoretic studies. The size of the node of a keyword reflects the weightage of the keyword in co-occurrence. The length between nodes shows the relationship strength between the nodes. As such, the shorter the length between nodes, the stronger the relationship between the nodes. The thickness of the line signifies the co-occurrences of the two keywords. The bolder the line, the higher the co-occurrences of the keywords [24].

Nodes with similar colors are categorized as one cluster. VOSviewer classified the 148 keywords into four clusters. The first cluster involves the red nodes with keywords such as antennas, channel capacity, cryptography, data privacy, decoding, estimation, fading channels, fisher information, gaussian distribution, information theory, Markov processes, mathematical models, Monte Carlo methods, network protocols, optimization, radar, sensors, set theory, Shannon entropy, stochastic systems, and topology. The second cluster involves the green nodes with keywords such as artificial intelligence, Bayesian networks, cluster analysis, computation theory, data mining, decision making, entropy, feature extraction, forecasting, iterative methods, Kullback Leibler divergence, learning algorithms, machine learning, probability density function, probability distributions, robotics, semantics, and uncertainty analysis. Blue nodes are the third cluster. They involve bioinformatics, computational biology, computer simulation, controlled study, information science, metabolism, neurons, physiology, and theoretical model. The last cluster is yellow and includes image analysis, image enhancement, image reconstruction, image segmentation, magnetic resonance imaging, and medical imaging.

The link strength is quantitative and can be used to identify the frequencies of co-occurrence. The total link strength involves all the link strengths with other nodes [23]. “Information theory” has the highest total link strength of 7598 and 2326 occurrences. Other nodes with high total link strengths include algorithms (2289), entropy (1488), and human (1445). Figure 3 shows the keyword co-occurrence map of information theoretic documents, while Table 7 presents the top 10 keywords with total link strengths.

**Figure 4:**
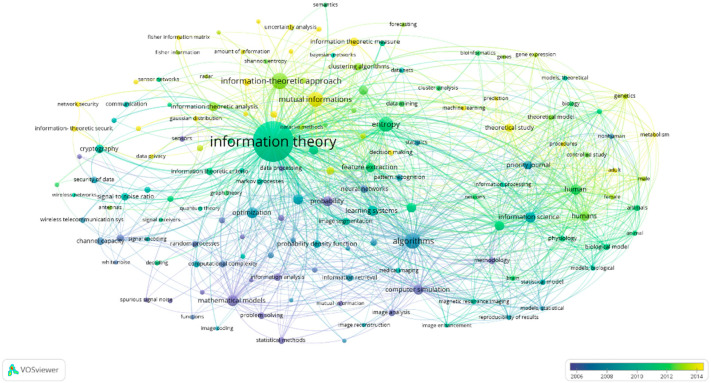
Overlay visualization map.

VOSviewer also provides an overlay visualization map to analyze keyword adoption across the years to observe the trend of the research title. Based on the overlay visualization map in Figure 4, the yellow node implies that the keyword is of current research interest. For example, current research trends in information theoretic focus on the information theoretic measure, network security, data privacy, fisher information matrix, uncertainty analysis, decision making, and machine learning. Based on these keywords, it can be forecast that future publications in information theoretic will revolve around the technological revolution to quantify and extract information from various signals. Moreover, researchers are studying to ensure useful and valuable information can be quantified while maintaining users’ and system security and privacy.

### 3.7. Citation Metrics

Table 8 tabulates the citation metrics of the 3701 documents retrieved as of 17 August 2022. There were 82,000 total citations with 22.16 citations per paper and an h-index of 116.

The most promising thematic lines in information theoretic include machine learning, robotics, quantification of information, and decision making. In Industry 4.0, all these thematic lines become the building blocks of transformation. Information theoretic is fundamental in processing and transmitting data and signals, which supports machine learning, robotics, information quantification, and decision making.

## 4. Discussion and Conclusions

This paper performed a comprehensive bibliometric analysis of the publications of information theoretic listed on the Scopus database from 1958 to 2022. The publication rate of information theoretic papers was low until the beginning of the 21st century. Publication growth has become steady, especially after 2003. Information theoretic has received much attention in computer science, engineering, and mathematics, using mathematical models and laws to quantify information in signals. The United States has the highest number of publications and received more than half of the total citations from all 3701 publications. The United States also has the highest scientific achievement after receiving the highest h-index. Even though China has the second highest number of publications, China could improve the quality of its contribution. The United States, United Kingdom, and China have the highest collaboration across countries. The source title that publishes the most information theoretic papers is Lecture Notes in Computer Science Including Subseries Lecture Notes in Artificial Intelligence and Lecture Notes in Bioinformatics by Springer Nature. The most cited publication is “Detection of signals by information theoretic criteria” by Wax and Kailath [25]. From the keyword analysis, the research interest in information theoretic has slowly shifted from mathematical models to technology-driven applications. The most promising thematic lines in information theoretic include machine learning, robotics, quantification of information and decision making.

Even though this paper has contributed to providing insights into the development of information theoretic publications since the first paper was listed in the Scopus database, this study has a limitation. This paper queried information theoretic documents based on title only. This analysis is very highly accurate at the time of the query. The Scopus database is continuously updating the new publications from time to time. Therefore, a bibliometric analysis of information theoretic may be revisited in a few years. Moreover, this bibliometric analysis extracts scientific data from the Scopus database only. Future studies may cover other databases for a more extensive understanding of information theoretic studies.

## Figures and Tables

**Table 1:** Document types of publications under information theoretic.

Document Types	Frequency	Percentage (%)
Conference Paper	1759	47.53
Article	1756	47.45
Book Chapter	72	1.95
Review	51	1.38
Erratum	18	0.49
Conference Review	14	0.38
Editorial	10	0.27
Letter	10	0.27
Note	6	0.16
Book	4	0.11
Data Paper	1	0.03
Total	3701	100

**Table 2:** Publication growth and citation metrics.

Year	TP	Percentage (%)	Cumulative Percentage (%)	NCP	TC	C/P	C/CP	h	g
1958	1	0.03	0.03	1	6	6.00	6.00	1	1
1964	1	0.03	0.05	1	8	8.00	8.00	1	1
1965	1	0.03	0.08	1	1	1.00	1.00	1	1
1966	1	0.03	0.11	1	19	19.00	19.00	1	1
1968	2	0.05	0.16	2	7	3.50	7.00	1	2
1969	2	0.05	0.22	1	3	1.50	3.00	1	1
1971	4	0.11	0.32	3	15	3.75	5.00	2	3
1972	3	0.08	0.41	2	38	12.67	19.00	2	3
1973	6	0.16	0.57	3	8	1.33	2.67	2	2
1974	9	0.24	0.81	8	547	60.78	68.38	7	9
1975	11	0.30	1.11	7	369	36.90	52.71	7	10
1976	14	0.38	1.49	13	305	21.79	23.46	8	14
1977	10	0.27	1.76	8	74	7.40	9.25	5	8
1978	10	0.27	2.03	10	416	41.60	41.60	8	10
1979	7	0.19	2.22	7	168	24.00	24.00	6	7
1980	6	0.16	2.38	5	47	7.83	9.40	4	6
1981	12	0.32	2.70	9	211	17.58	23.44	6	12
1982	10	0.27	2.97	5	123	12.30	24.60	3	10
1983	21	0.57	3.54	15	154	7.33	10.27	6	12
1984	18	0.49	4.03	12	311	17.28	25.92	8	17
1985	16	0.43	4.46	12	2934	183.38	244.50	6	16
1986	9	0.24	4.70	6	126	14.00	21.00	3	9
1987	19	0.51	5.21	15	358	18.84	23.87	6	18
1988	14	0.38	5.59	10	270	19.29	27.00	7	14
1989	18	0.49	6.08	13	488	14.79	37.54	5	18
1990	12	0.32	6.40	11	554	46.17	54.36	6	12
1991	15	0.41	6.81	13	1197	79.80	92.08	7	15
1992	21	0.57	7.38	15	1758	83.71	117.20	8	21
1993	34	0.92	8.30	30	1102	32.41	36.73	13	33
1994	18	0.49	8.78	12	936	52.00	78.00	5	18
1995	23	0.62	9.40	21	467	20.30	22.24	10	21
1996	24	0.65	10.05	20	989	41.21	49.45	10	24
1997	29	0.78	10.83	23	1151	39.69	50.04	12	29
1998	45	1.22	12.05	37	3202	71.16	86.54	19	45
1999	37	1.00	13.05	33	995	26.89	30.15	14	31
2000	48	1.30	14.35	40	2200	45.83	55.00	19	46
2001	50	1.35	15.70	41	2007	40.14	48.95	19	44
2002	66	1.78	17.48	54	2575	39.02	47.69	20	50
2003	101	2.73	20.21	87	5287	52.35	60.77	30	72
2004	87	2.35	22.56	74	1592	18.30	21.51	20	38
2005	137	3.70	26.26	121	4019	29.34	33.21	34	60
2006	137	3.70	29.96	115	3301	24.09	28.70	28	55
2007	157	4.24	34.21	133	6070	38.91	45.64	31	76
2008	143	3.86	38.07	115	4410	30.84	38.35	26	65
2009	166	4.49	42.56	140	7136	42.99	50.97	35	83
2010	194	5.24	47.80	164	4501	23.20	27.45	32	63
2011	170	4.59	52.39	139	2484	14.61	17.87	26	44
2012	151	4.08	56.47	128	3308	22.05	25.84	27	54
2013	178	4.81	61.28	156	2843	15.97	18.22	30	48
2014	130	3.51	64.79	109	1492	11.48	13.69	21	34
2015	169	4.57	69.36	144	2119	12.54	14.72	24	38
2016	161	4.35	73.71	127	1667	10.35	13.13	22	34
2017	169	4.57	78.28	146	1706	10.09	11.68	21	33
2018	171	4.62	82.90	145	1660	9.71	11.45	21	32
2019	183	4.94	87.84	154	1162	6.35	7.55	16	25
2020	170	4.62	92.46	135	760	4.47	5.63	13	19
2021	184	4.97	97.43	95	327	1.78	3.44	7	12
2022	96	2.57	100.00	16	17	0.18	1.06	1	1
Total	3701	100.00			82,000				

**Table 3:** Subject Areas.

Subject Area	TP	Percentage
Computer Science	2259	32.89
Engineering	1254	18.26
Mathematics	1068	15.55
Physics and Astronomy	574	8.36
Social Sciences	248	3.61
Materials Science	190	2.77
Biochemistry, Genetics, and Molecular Biology	183	2.66
Chemistry	128	1.86
Medicine	128	1.86
Decision Sciences	121	1.76
Neuroscience	118	1.72
Arts and Humanities	82	1.19
Agricultural and Biological Sciences	80	1.16
Earth and Planetary Sciences	69	1.00
Environmental Science	58	0.84
Economics, Econometrics, and Finance	54	0.79
Business, Management, and Accounting	47	0.68
Psychology	46	0.67
Multidisciplinary	44	0.64
Energy	34	0.49
Chemical Engineering	32	0.47
Health Professions	21	0.31
Immunology and Microbiology	14	0.20
Pharmacology, Toxicology, and Pharmaceutics	14	0.20
Nursing	3	0.04
Chemical Engineering	32	0.47

**Table 4:** Top 10 geographical contribution of information theoretic publications.

Country	TP	NCP	TC	C/P	C/CP	h-Index	g-Index
United States	1624	1371	45,797	28.20	33.40	89	181
China	285	221	3152	11.06	14.26	28	46
United Kingdom	279	242	7352	26.35	30.38	40	78
Germany	193	161	4161	21.56	25.84	35	60
Canada	191	158	4634	24.26	29.33	33	64
Japan	181	136	3032	16.75	22.29	25	52
Italy	173	151	4272	24.69	28.29	28	63
India	146	112	1172	8.03	10.46	17	29
Australia	126	111	3964	31.46	35.71	25	61
Israel	113	95	4699	41.58	49.46	32	68

**Table 5:** Top 10 source titles of information theoretic publications.

Source Title	TP	Percent	TC	Publisher	Cite Score 2021	SJR2021	SNIP2021	h
Lecture Notes In Computer Science Including Subseries Lecture Notes In Artificial Intelligence And Lecture Notes In Bioinformatics	221	5.97	4163	Springer Nature	2.1	0.407	0.534	415
IEEE International Symposium On Information Theory Proceedings	139	3.76	1050	IEEE	2.5	0.872	0.676	91
IEEE Transactions On Information Theory	103	2.78	8382	IEEE	6.2	1.731	1.735	279
Proceedings Of SPIE The International Society For Optical Engineering	84	2.27	365	SPIE	0.9	0.184	0.239	179
Entropy	70	1.89	498	Multidisciplinary Digital Publishing Institute (MDPI)	4.4	0.553	1.084	81
ICASSP IEEE International Conference On Acoustics Speech And Signal Processing Proceedings	56	1.51	370	IEEE	5.8	1.229	1.178	163
IEEE Transactions On Signal Processing	30	0.81	1454	IEEE	10.5	2.682	2.109	275
Advances In Neural Information Processing Systems	29	0.78	481	N/A	N/A	N/A	N/A	266
Proceedings Of The International Joint Conference On Neural Networks	29	0.78	139	IEEE	2.3	0.51	0.605	82
IEEE Access	22	0.59	68	IEEE	6.7	0.927	1.326	158

**Table 6:** Top 10 most cited publications.

Author	Title	Year	Cites	Cites Per Year	Source Title
M. Wax, T. Kailath [25]	Detection of signals by information theoretic criteria	1985	2731	73.81	IEEE Transactions on Acoustics, Speech, and Signal Processing
A. Goldsmith, S.A. Jafar, I. Maric, S. Srinivasa [27]	Breaking spectrum gridlock with cognitive radios: An information theoretic perspective	2009	1970	151.54	Proceedings of the IEEE
E. Biglieri, J. Proakis, S. Shamai [28]	Fading channels: Information-theoretic and communications aspects	1998	1462	60.92	IEEE Transactions on Information Theory
J.V. Davis, B. Kulis, P. Jain, S. Sra, I.S. Dhillon [29]	Information-theoretic metric learning	2007	1456	97.07	24th International Conference on Machine Learning, ICML 2007
M. Bloch, J. Barros, M.R.D. Rodrigues, S.W. McLaughlin [30]	Wireless information-theoretic security	2008	1418	101.29	IEEE Transactions on Information Theory
T.P. Pedersen [31]	Non-interactive and information-theoretic secure verifiable secret sharing	1992	1329	44.3	11th Conference on Advances in Cryptology, CRYPTO 1991
N.X. Vinh, J. Epps, J. Bailey [32]	Information theoretic measures for clusterings comparison: Variants, properties, normalization, and correction for chance	2010	1121	93.42	Journal of Machine Learning Research
I.S. Dhillon, S. Mallela, D.S. Modha [33]	Information-theoretic co-clustering	2003	919	48.37	9th ACM SIGKDD International Conference on Knowledge Discovery and Data Mining, KDD ‘03
L.H. Ozarow, S. Shamai, A.D. Wyner [34]	Information Theoretic Considerations for Cellular Mobile Radio	1994	841	30.04	IEEE Transactions on Vehicular Technology
G. Brown, A. Pocock, M.-J. Zhao, M. Lujan [35]	Conditional likelihood maximisation: A unifying framework for information theoretic feature selection	2012	828	82.8	Journal of Machine Learning Research

**Table 7:** Top 10 keywords of information theoretic publications.

Keywords	Occurrences	Total Link Strengths
Information theory	2326	7598
Algorithms	365	2289
Entropy	327	1488
Human	164	1445
Information Science	163	1382
Information-theoretic approach	391	1371
Humans	137	1360
Algorithm	128	1288
Mutual information	320	1274
Computer simulation	149	1070

**Table 8:** Citation metrics.

Items	Data
Date of Extraction	17 August 2022
Papers	3701
Citations	82,000
Years	64 (1958–2022)
Citation/Year	1281.25
Citation/Paper	22.16
Citation/Author	36,723.02
Papers/Author	1694.61
Authors/Paper	2.82
h-index	116
g-index	221
